# Hemocompatibility of Nanotitania-Nanocellulose Hybrid Materials

**DOI:** 10.3390/nano11051100

**Published:** 2021-04-24

**Authors:** Fredric G. Svensson, Vivek Anand Manivel, Gulaim A. Seisenbaeva, Vadim G. Kessler, Bo Nilsson, Kristina N. Ekdahl, Karin Fromell

**Affiliations:** 1 Department of Molecular Sciences, Swedish University of Agricultural Sciences, P.O. Box 7015, SE-750 07 Uppsala, Sweden; fredric.svensson@slu.se (F.G.S.); gulaim.seisenbaeva@slu.se (G.A.S.); vadim.kessler@slu.se (V.G.K.); 2 Rudbeck Laboratory C5:3, Department of Immunology, Genetics and Pathology, Uppsala University, SE-751 85 Uppsala, Sweden; vivekanand.manivel@igp.uu.se (V.A.M.); bo.nilsson@igp.uu.se (B.N.); kristina.nilsson_ekdahl@igp.uu.se (K.N.E.); 3 Linnæus Centre for Biomaterials Chemistry, Linnæus University, SE-391 82 Kalmar, Sweden

**Keywords:** nanocellulose, titania, coagulation, platelets, complement system, contact system

## Abstract

In order to develop a new type of improved wound dressing, we combined the wound healing properties of nanotitania with the advantageous dressing properties of nanocellulose to create three different hybrid materials. The hemocompatibility of the synthesized hybrid materials was evaluated in an in vitro human whole blood model. To our knowledge, this is the first study of the molecular interaction between hybrid nanotitania and blood proteins. Two of the hybrid materials prepared with 3 nm colloidal titania and 10 nm hydrothermally synthesized titania induced strong coagulation and platelet activation but negligible complement activation. Hence, they have great potential as a new dressing for promoting wound healing. Unlike the other two, the third hybrid material using molecular ammonium oxo-lactato titanate as a titania source inhibited platelet consumption, TAT generation, and complement activation, apparently via lowered pH at the surface interface. It is therefore suitable for applications where a passivating surface is desired, such as drug delivery systems and extracorporeal circuits. This opens the possibility for a tailored blood response through the surface functionalization of titania.

## 1. Introduction

Biomaterials are used in a wide range of different healthcare applications, from implants to dialysis membranes and cell carriers. Today, the trend is towards new types of biomaterials, which can be designed with specific properties to achieve unique functionalities. An important example is in the field of wound healing, as wound management is critical to both preventing infection of the wound and promoting tissue regeneration. The traditional wound dressings such as gauzes and bandages are considered passive dressings and only physically protect the wound, neither protecting against infection nor promoting healing [1]. There is great interest in developing active wound dressings, e.g., dressing that protects against infection and/or stimulates the healing processes. Particularly, nanomaterials, including metal/metal oxide nanoparticles, have demonstrated healing properties with their ability to initiate immune responses, which subsequently activate tissue reparation processes [2,3,4]. Gels based on biopolymers such as alginate and nanocellulose have received considerable attention for application in wound dressing as they facilitate a moist environment, gas exchange, removal of wound exudate, and easy application and possess a fibrous structure that can function as a scaffold for improved tissue regeneration [5,6,7,8,9].

Important for all types of biomaterial implants is their biocompatibility, defined by Williams [10] as “The ability of a material to perform with an appropriate host response in a specific application”. Many of the materials, however, are far from ideal, leading to low quality of life for patients and high costs for society. Innate immunity is essential for the defense against microorganisms and foreign substances, including biomaterials, and regulates the initial discrimination between self- and non-self structures in the human body. In the blood, the innate immune system consists of several cascade systems that include the complement, contact, coagulation, and fibrinolytic systems, together with cellular defense systems such as different populations of leukocytes, platelets, and endothelial cells.

When a biomaterial comes in contact with blood, it is immediately covered with plasma proteins. The composition of this protein layer dictates how the biomaterial is perceived in the body. Fibrinogen is one of the primary proteins initially adsorbed to the material surface and together with other coagulation factors mediates platelet adhesion and activation [11]. Subsequently, the primary proteins are partially replaced by others including those belonging to the intrinsic pathway of coagulation, i.e., Factor (F)XII and its substrates FXI and Prekallikrein (PK). When FXII binds to the surface, it is activated, which through a series of events commencing with activation of FXI leads to activation of prothrombin to thrombin. Thrombin cleaves fibrinogen to fibrin which polymerizes and forms a clot. Thrombin also serves as a platelet agonist leading to further platelet activation and aggregation. Activated platelets further prompt the coagulation cascade and thereby constitute an efficient amplification loop [12]. In addition, if FXII instead activates its second substrate PK, the result is generation and release of the peptide bradykinin, which has very potent proinflammatory and proangiogenic properties [13].

The complement system can also be activated by contact with artificial surfaces, mainly via the classical pathway or the alternative pathway, which leads to the generation of the anaphylatoxins C3a and C5a, which promote leukocyte activation and pro-inflammatory response [13,14]. In addition, the larger cleavage fragment of C3 and C3b is deposited on the surface and acts as an opsonin as well as the basis for formation of additional convertases. This results in subsequent activation of C5 to the anaphylatoxin C5a and formation of the terminal sC5b-9 complex [15]. Besides platelet, coagulation, and complement activation, leukocytes (i.e., neutrophils and monocytes) are also recruited to the foreign material surface and become activated leading to acceleration of the inflammatory response [16]. However, since biocompatibility is a complex process that involves both the material and the tissue with which it should interact [17], these reactions, which may result in fatal consequences for many types of biomaterials, specifically those intended for direct blood contact, are of utmost importance during the wound healing process [18,19].

Nanocellulose is the nanosized form of cellulose, one of the most abundant biomolecules on Earth [20]. It consists of linear polymers of glucose, covalently bonded by β(1–4) linkages. Nanocellulose is mostly derived from plant (e.g., forestry residues) or bacterial sources and can be divided into three sub-groups: cellulose nanocrystals (CNCs), cellulose nanofibrils (CNFs), and bacterial nanocellulose (BC) [5]. CNCs have dimensions between 5 and 20 nm to 100 and 500 nm with a rather high degree of crystallinity. CNFs consist of micrometer long cellulose fibrils with typical diameters of 20–40 nm [21], while BC has dimensions similar to CNF. BC is obtained directly as pure cellulose [5]. In recent years, nanocellulose has been investigated for new potential applications such as drug delivery [22,23,24] and nanopaper electronic devices [25]. Nanocellulose can be produced from residuals from the forest industry thereby increasing the utilization rate. In addition to nanocellulose, other biopolymers including chitosan [26] and alginate [27] have been investigated for controlled delivery of antibiotics.

Nanocellulose-nanotitania composite films are attractive materials for controlled drug release [23,28]. These materials can be considered as type 2 organic-inorganic hybrid materials according to the definition proposed by Sanchez [29] because they are produced via chemical grafting of nanotitania, exploiting surface complexation with deliberately introduced carboxylic functions. Functional groups of drug molecules are adsorbed to the surface of nanotitania and slowly released, compared to addition of drugs to pure nanocellulose [23,28,30]. A hybrid material of chitosan containing 0.25% titania showed improved cell proliferation and survival for mouse fibroblasts and antibacterial properties towards *Staphylococcus aureus* [31]. Although commonly regarded as inert, titania has been proven to activate the contact factor pathway of the coagulation system, resulting in thromboinflammation, even at as low concentrations of 50 ng/mL when added to whole human blood [32].

Our approach was to design a material that optimally modulates the hemostatic reactions and the immune system and thereby constitutes a platform to accelerate wound healing. Recently, we investigated sprays containing nanosized colloidal titania particles applied to burn wounds in vivo on rats. The wounds treated with titania nanoparticles exhibited improved healing and tissue regeneration compared to untreated controls. The wound-healing effect of titania was ascribed to activation of the contact system demonstrated as generation of FXIIa-antithrombin (AT) and FXIIa–C1inhibitor (C1INH) complexes and thrombin–antithrombin (TAT) formation [3]. Since the contact system activation also leads to subsequent formation of proangiogenic bradykinin, in addition to the coagulative effect, it may be assumed that this activation product has a role in initiation of the observed healing process.

By uniting the promising wound-dressing properties from nanocellulose and the potential of titania to stimulate initiation of wound healing processes, we sought to produce hybrid materials that combine the two effects into one material. However, for clinical use, the blood compatibility of the materials needs to be investigated. Therefore, we studied the interaction between human whole blood and our three different hybrid materials using (i) ca. 3 nm colloidal titania [33], (ii) ca. 10 nm hydrothermally synthesized titania [34], and (iii) molecular ammonium oxo-lactato titanate as sources [35] of titania dispersed in cellulose nanofibrils.

## 2. Materials and Methods

### 2.1. Syntheses and Characterization of Materials

Carboxymethylated fibrillar nanocellulose (CNF, 0.54 wt.% aqueous suspension) was provided by Innventia AB (Stockholm, Sweden). Three sources of titania were used: in-house hydrothermally synthesized titania (ca. 10 nm anatase from titanium(IV) ethoxide, Sigma-Aldrich, Sweden ), Captigel (colloidal titania, 2–3 nm, Captigel AB, Uppsala, Sweden), and TiBALDH (Sigma Aldrich, Sweden for in situ formation of titania). TiBALDH has been reported to produce TiO_2_ (anatase) particles of ca. 3.2 nm [35].

Poly(ethylene)glycol (PEG, Aldrich, 35,000 Da) was added to improve the mechanical properties of the materials, being considered a biocompatible polymer [36]. These materials are abbreviated as CNF_PEG_TiO_2_, CNF_PEG_Captigel, and CNF_PEG_TiBALDH, respectively. One material synthesized without titania, CNF_PEG, served as a control.

Hybrid materials of 3:1 (*w/w*, dry weight) CNF/TiO_2_ were synthesized. The appropriate amount of titania was added under magnetic stirring to a CNF suspension pre-heated to 70 °C with 2 mL 1 wt.% poly(ethylene)glycol (PEG) and was stirred for 2 h. The hydrothermally synthesized titania was sonicated for 10 min in 2 mL dH_2_O prior to addition. Prior to addition, TiBALDH was diluted in 2 mL dH_2_O, and Captigel was diluted in 2 mL ethanol (95%, Solveco AB, Sweden). The suspensions were then poured into Petri dishes and kept at ca. 40 °C until dry. The materials were then kept cool and dark prior to the exposure to blood.

### 2.2. Characterization of Materials

The morphology of the synthesized hybrid materials was characterized by scanning electron microscopy (SEM) (Hitachi TM-1000) coupled with energy-dispersive X-ray analysis (EDX) (Hitachi High Tech in Europe, Stockholm, Sweden) for elemental analysis.

### 2.3. Heparinization of Materials for Whole Blood Model Studies

All materials in contact with blood apart from the composite materials under investigation (i.e., incubation chambers, pipette tips, Falcon tubes, rubber tubing, etc.) were covered with a double layer of Corline heparin conjugate according to the manufacturer’s procedure (Corline Systems AB, Uppsala, Sweden) to avoid surface activation of the coagulation and complement systems.

### 2.4. Human Whole Blood Model

**Figure A1:**
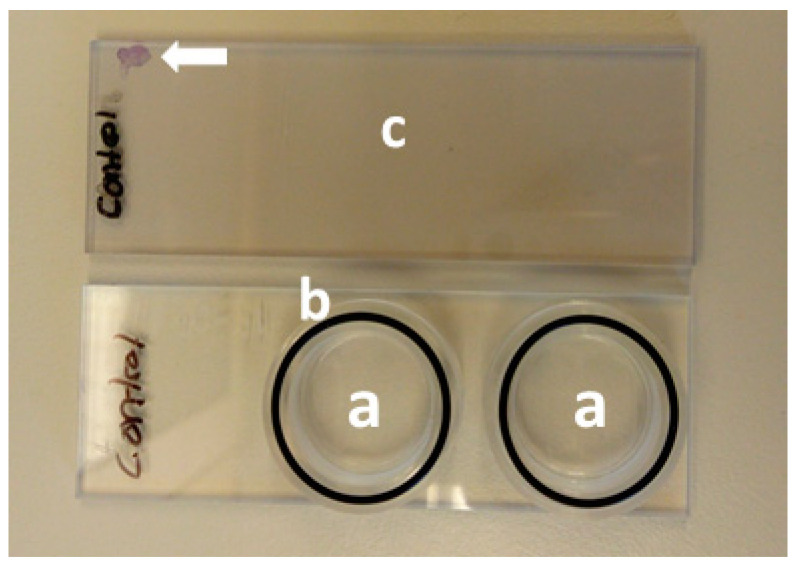
Incubation chamber with two wells (**a**), rubber tightening (**b**), and lid (**c**). Toluidine blue was applied on the surface to confirm successful heparinization. The toluidine blue dye binds to heparin causing purple-blue staining (indicated by the white arrow) resistant to removal upon washing with water.

To investigate the effect of the composite materials on human blood, the slide chamber model was used [37]. Human whole blood was collected from five healthy blood donors (3 males and 2 females), who not had received any medication during the last 10 days. Ethical approval was obtained from the regional ethics committee in Uppsala (#2008-264). Blood was collected immediately before the experiments, and all composite materials were tested in duplicate for each donor. To attenuate coagulation activation during the experiments, a low amount of heparin (0.5 units/mL blood; Leo Pharma, Malmö, Sweden) was added directly after blood collection. To each well (see Figure A1, Appendix A), 1.5 mL whole blood was added, and the hybrid materials were placed in the well with a top lid held together by clamps. A Corline heparinized surface (CHS, i.e., the lid coated with heparin) was used as a control to evaluate the blood activation induced by the system without the presence of the test materials. The whole blood was incubated with the four different materials and the CHS control at 37 °C in a rotating device for 1 h. After incubation, 1 mL of blood from each well was transferred into Eppendorf tubes containing EDTA (10 mM final concentration) to stop the activation of the complement and coagulation cascades. Platelet counts were performed on the initial blood samples as well as on the blood after incubation in the chambers using a Sysmex XP-300 Hematology Analyzer (Sysmex Corp. Kobe, Japan). The number of platelets remaining in the blood samples after the different treatments was normalized against initial platelet number for each blood donor and presented as fraction of remaining platelets relative to the initial value. The samples were centrifuged at 2575× *g* for 15 min at 4 °C, and the plasma was collected and stored at −80 °C until analyzed.

### 2.5. Enzyme-Linked Immunosorbent Assay (ELISA) for Coagulation and Complement Markers

A sandwich ELISA was used to assess the levels of complement, coagulation, and contact system activation markers. In all the in-house ELISAs described here (unless otherwise specified), capture antibodies were diluted in phosphate buffer saline (PBS). The working buffer (for blocking and sample dilution) contained PBS with 1% bovine serum albumin, 0.05% Tween-20, and 10 mM EDTA, and for washing PBS + 0.05% Tween-20 was used. As substrate for the conjugates with horseradish peroxidase (HRP), 3,3′5,5′-tetramethylbenzidine (TMB) was used, and this reaction was stopped by the addition of 1 M H_2_SO_4_. The absorbance was measured by spectrometry at 450 nm. Specific details for each assay are found below.

### 2.6. C3a and sC5b-9 ELISA

C3a levels were determined using monoclonal antibody (mAb) 4SD17.3 for capture and biotinylated rabbit polyclonal anti-C3a antibody (pAb) Rb-a-Hu C3a-biotin followed by SA-HRP (GE Healthcare, RPN1231V) for detection. In order to analyze the amount of released sC5b-9, mAb anti-neoC9 (Diatec Monoclonals AS, Norway) was used for capture and anti-C5 pAb (Biosite BP373) followed by SA-HRP (GE Healthcare, RPN1231V) for detection. Standard solutions were prepared from Zymosan activated serum calibrated against commercially available kits (MicroVue, Quidel Corp., Santa Clara, CA, USA). C3a and sC5b-9 values were measured as µg/L and then normalized against the initial values and presented as relative amount to the initial value.

### 2.7. TAT ELISA

The TAT complexes were captured by anti-human thrombin pAb and detected by HPR-conjugated anti-human antithrombin pAb, both from Enzyme Research laboratories, South Bend, IN, USA. As standard pooled human serum diluted in working buffer was used for quantification. Results are presented as µg/L.

### 2.8. FXIa–C1INH and FXIIa–C1INH ELISA

To measure the levels of contact activation complexes by ELISA, either polyclonal goat (anti-human FXII or anti-human FXI antibodies) (Enzyme research Laboratories, South Bend, IN, USA) was used as capture agent along with a biotinylated rabbit anti-human C1INH pAb for detection (prepared in-house) followed by incubation with streptavidin-HRP for detection. Standards were prepared from complexes with purified FXIIa–C1INH and FXIa–C1INH (using a molar excess of C1INH) diluted in freshly drawn lepirudin plasma. Results are expressed as arbitrary units (AU)/mL.

### 2.9. Statistical Analyses

A two-sided two-sample t-test was used to test for differences among groups using the R program for statistical computing [38].

## 3. Results

### 3.1. Characterization of the Materials

**Figure 1:**
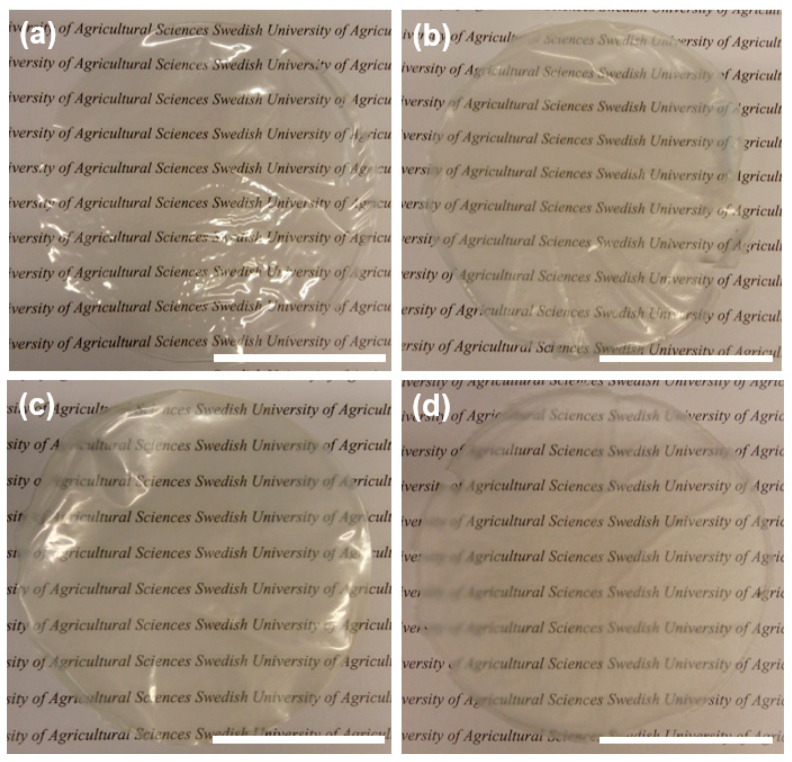
Photographs of the newly synthesized materials. Bright areas are light reflections. (**a**) CNF_PEG, (**b**) CNF_PEG_TiO_2_, (**c**) CNF_PEG_Captigel, and (**d**) CNF_PEG_TiBALDH. Scale bars indicate 4 cm.

**Figure 2:**
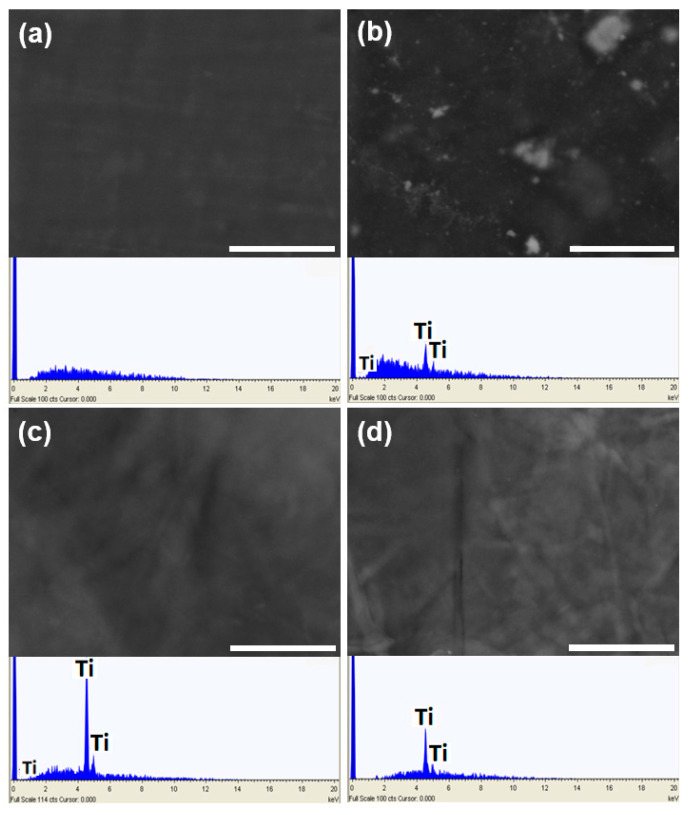
SEM micrographs of the control material, (**a**) CNF_PEG, and the three hybrid materials (**b**) CNF_PEG_TiO_2_, (**c**) CNF_PEG_Captigel, and (**d**) CNF_PEG_TiBALDH. The inserted EDX spectra beneath the micrographs confirm the presence of titanium in materials (**b**–**d**). The scale bars indicate 10 μm.

The synthesized materials have good strength and flexibility as well as high optical transparency as illustrated in the photographs in Figure 1. The materials could be stored in MilliQ-water for up to two weeks without disintegration, although their strength dramatically decreased. SEM-EDX analyses (Figure 2) of CNF_PEG and CNF_PEG_Captigel revealed very smooth and homogenous surfaces, while the CNF_PEG_TiBALDH surface was rougher, even though the titania distribution still appeared to be homogenous. However, the CNF_PEG_TiO_2_ material showed aggregates of titania unevenly distributed over the surface, and aggregates inside the material were covered with nanocellulose. The stable titania colloid in Captigel was better dispersed in the nanocellulose suspension, forming a more homogenous material. The hybrid materials had a thickness of between 10 and 20 µm, while the reference material (CNF_PEG) was thinner and had a thickness of about 4 µm.

### 3.2. Platelet Activation

**Figure 3:**
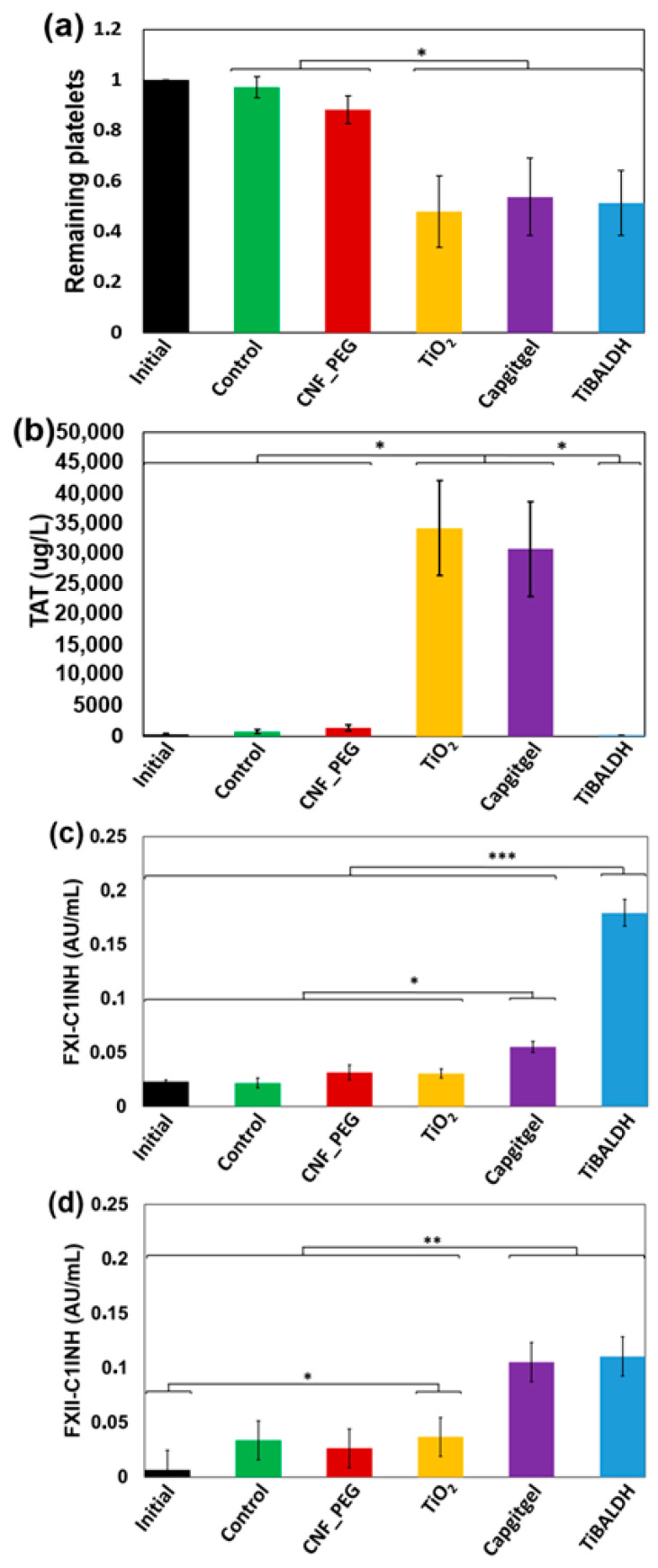
Assessment of coagulation activation induced by the test surfaces (CNF_PEG_TiO_2_, CNF_PEG_Captigel, and CNF_PEG_TiBALDH, abbreviated to TiO_2_, Captigel, and TiBALDH, respectively, in the figure) and the controls (CHS and CNF_PEG) compared to the initial samples after incubation in human whole blood. (**a**) Remaining platelets (n = 5), (**b**) generation of TAT-complexes (n = 5). (**c**) Formation of FXIa–C1INH complexes (n = 3) and (**d**) of FXIIa–C1INH complexes (n = 3). Data are presented as mean ± SEM. The data in (a) were normalized against the initial values for each donor. * Significant at *p* < 0.05 level, ** significant at *p* < 0.01 level, *** significant at *p* < 0.001 level.

**Figure A2:**
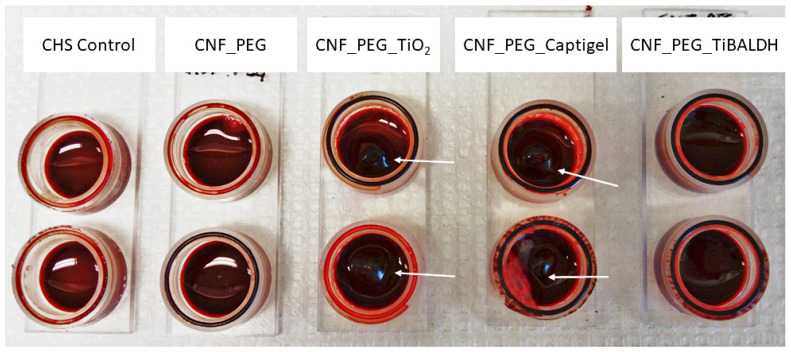
Blood chambers after incubation for the control and the four different materials. The white arrows indicate blood clots.

The relative number of platelets remaining in the blood after incubation with the test surfaces and the controls was measured and normalized against the initial blood sample from respective donors (Figure 3). Normalization was carried out because the variation in platelet count varies greatly between different blood donors (normal range: 150–400 × 10^9^ platelets/L). After incubation with blood, visible clots were observed on the CNF_PEG_TiO_2_ and CNF_PEG_Captigel materials, (Figure A2), which also resulted in a large reduction in platelets in these blood samples but not on CNF_PEG_TiBALDH. Some samples nevertheless showed a reduction in platelet count. This seems to be an individual response as three donors showed strong reduction of platelets, and the two other showed very little reduction. The CNF_PEG and the CHS control both had an unchanged number of platelets compared to the initial sample. Although there were some variances in response between different donors, the titania-containing samples (i.e., CNF_PEG_TiO_2_, CNF_PEG_Captigel, and CNF_PEG_TiBALDH) generally induced activation of the platelets.

### 3.3. TAT Complexes

The generation of thrombin is one of the cornerstones of the coagulation cascade, as it cleaves fibrinogen to fibrin resulting in clot formation. However, measuring thrombin to monitor the coagulation process is difficult, since it is rapidly inactivated by the serine protease inhibitor (SERPIN) AT forming TAT complexes. The advantage is that these complexes are very stable and therefore more suitable parameters to follow coagulation activation. Measuring TAT levels has therefore become a well-established method to monitor coagulation activation [39,40]. The TAT levels were measured by ELISA in all samples after blood exposure to the four different materials, the CHS control, and in the initial blood sample collected before onset of the experiment (Figure 3). The levels varied somewhat between different blood donors (as reflected by the large standard deviations), but the overall result clearly showed that CNF_PEG_TiO_2_ and CNF_PEG_Captigel generated significantly higher TAT levels compared to CNF_PEG and CNF_PEG_TiBALDH and the CHS control surface as well as the initial samples. The crystalline phase of titania (anatase or rutile) has been reported to affect its cytotoxicity [41]. However, in case of the CNF_PEG_TiO_2_ materials, the titania particles are heavily aggregated, and the effects may be more dependent on aggregate sizes and shapes rather than crystalline phase. Interestingly, TAT levels for CNF_PEG_TiBALDH were always very low, and no clots were observed. This was suspected to be a cause of residual lactic acid from the TiBALDH precursor as it has been reported that coagulation is inhibited by a decrease in pH caused by lactic acid [42]. To investigate this, a few drops of 0.9% NaCl were added on top of the materials, and then the pH was measured at the surface interfaces using a piece of pH paper within 5–10 s. This revealed an acidic response (pH ca. 6) for CNF_PEG_TiBALDH, while the other two materials demonstrated a neutral response (pH ca. 7 or slightly above) suggesting that there is a pH difference at the interface between blood and the three surfaces.

### 3.4. Contact Activation Complexes

To further investigate the mechanisms behind the coagulation activation triggered by the CNF_PEG modified materials, C1INH complexes with FXIa and FXIIa were measured (Figure 3c,d). Upon contact with certain foreign surfaces, FXII is activated and in turn activates FXI, which eventually results in cleavage of prothrombin into thrombin, which initiates clot formation. Just like thrombin, the activated forms of both FXII (FXIIa) and FXI (FXIa) form stable complexes with the SERPINS AT and C1INH that can be analyzed with ELISA. Some samples in this study had very high levels of TAT, which potentially can interfere with the measurement of other AT-complexes; therefore, the FXIIa–C1INH and FXIa–C1INH complexes were measured instead. The levels of FXIIa–C1INH and FXIa–C1INH complexes were very low for the CHS control surface and the CNF_PEG.

Surprisingly, they were also low in the blood samples that had been in contact with CNF_PEG_TiO_2_ and CNF_PEG_Captigel, which clearly triggered clot formation. An exception was CNF_PEG_TiBALDH, which resulted in elevated levels of both FXIIa– C1INH and FXIa–C1INH and in one instance where FXIIa–C1INH concentration from the CNF_PEG_Captigel was elevated. We have previously seen that C1INH binds the fibrin in the clots (Fromell et al., manuscript in preparation).

This may reduce their availability both as single molecules and in complex with proteases in the blood plasma, which would explain the low measured levels in the clotted samples. This hypothesis was reinforced by the relatively high levels in the TiBALDH samples. In contrary to the CNF_PEG_TiO_2_ and CNF_PEG_Captigel, these surfaces did not induce any clotting but still had the potential to trigger contact activation, which made the formed complexes available for detection by ELISA as they were not captured in the clots.

### 3.5. Complement Activation

**Figure 4:**
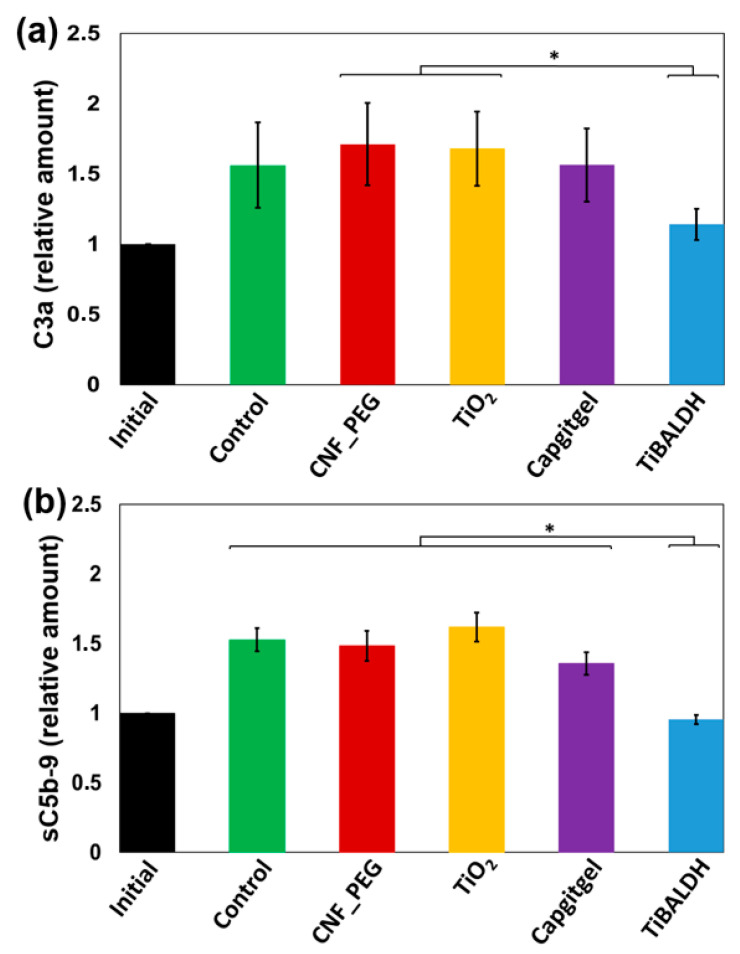
Generation of complement activation products after incubation of the test surfaces, (CNF_PEG_TiO_2_, CNF_PEG_Captigel, and CNF_PEG_TiBALDH, abbreviated to TiO_2_, Captigel, and TiBALDH, respectively, in the figure) and the controls (CHS and CNF_PEG) compared to the initial samples after incubation in human whole blood. (**a**) Relative concentration of C3a (n = 3). (**b**) Relative concentrations of sC5b-9 (n = 3). Data are presented as mean ± SEM. Significant differences (*p* < 0.05) are indicated by *. The data in (**a**,**b**) were normalized against the initial value of each donor.

The C3a concentrations after incubating the blood in the chamber model were measured and found to be slightly higher compared to initial sample (Figure 4a), but the different surfaces did not differ to any great extent or in comparison with the CHS control. The increased generation of C3a in all samples including the controls is due to the air bubble that inevitably forms between the blood and the chamber lid, which initiates a low level of complement activation. Interestingly, the lowest C3a levels were measured in the plasma samples that had been in contact with CNF_PEG_TiBALDH materials. In those samples, the levels were in principle at the same low level as the initial sample, despite the fact that also the CNF_PEG_TiBALDH surfaces were incubated with the same chamber model as the other samples, including the activating air bubble. The sC5b-9 concentrations (Figure 4b) were low and approximately the same for the control, CNF-PEG, CNF_PEF_TiO_2_, and CNF_PEG_Captigel after incubation. Again, the CNF_PEG_TiBALDH, demonstrated significantly (*p* < 0.05) lower quantities of generated complement activation markers compared to the other materials and control.

## 4. Discussion

In the present work we synthesized three novel nanotitania-nanocellulose hybrid materials for human use and evaluated their hemocompatibility by assessing the material-induced activation of the coagulation system, complement system, and contact system. Cellulose nanofibrils were mixed with nanotitania in an effort to combine the wound-dressing properties of CNF and the wound healing properties of nanotitania marked by the initiation of coagulation and complement activation. Homogenous hybrid materials were obtained for colloidal titania (Captigel) and the molecular precursor TiBALDH with the CNF suspension. Aggregation of the pre-synthesized, non-stabilized titania in the CNF suspension instead led to formation of large titania aggregates. This highlights the benefits of exploiting molecular/colloidal starting materials to obtain homogenous hybrid materials.

The strongest coagulation response was achieved with the CNF_PEG_TiO_2_ and CNF_PEG_Captigel materials, both leading to thrombin formation and platelet activation resulting in pronounced clot formations. The formed blood clot, constituted of platelets embedded in a fibrin network, is an optimal platform to improve wound healing as we have demonstrated earlier [3].

In previous studies using TiO_2_ nanoparticles and solid titanium substrates, respectively, we have demonstrated that coagulation activation was initiated via the FXII, of the contact system [32,37]. Therefore, the hypothesis was that the same mechanisms initiate the activation on the surfaces in the present study. Since high TAT levels associated with strong coagulation in in vitro samples may affect the measurement of other AT complexes (e.g., FXIIa-AT and FXIa-AT), we decided to measure the corresponding C1INH complexes instead. Somewhat unexpectedly, only the CNF_PEG_TiBALDH surface generated elevated plasma levels of the contact activation complexes (FXIIa–C1INH and FXIa–C1INH), i.e., the only one of the three titania-modified surfaces that did not trigger any coagulation activation. The other two highly thrombogenic surfaces (CNF_PEG_TiO_2_ and CNF_PEG_Captigel) showed no or very low levels of the C1INH complexes. However, significantly lower concentrations of C1INH complexes compared to the corresponding AT-complexes have also been measured on previous occasions. One explanation is that C1INH both free and in complex has a strong tendency to bind fibrin in the formed clots. It is therefore likely that coagulation nevertheless was initiated via the intrinsic pathway, although no C1INH complexes could be detected.

The low levels of C3a and sC5b-9 indicated that the complement system had not been activated. Platelet and coagulation activation is the first reaction that occurs both to stop bleeding and in response to contact-activating material, resulting in clot formation. It is therefore likely that complement activation starts somewhat later in the inflammatory phase and for that reason did not reach measurable levels during the initial stage evaluated in this study. It is also an advantage that the present materials do not trigger an immediate and extremely strong inflammatory response.

The CNF_PEG_TiBALDH material turned out to have completely different properties compared to the other two hybrid materials. Despite some variation in platelet reduction, this surface showed no tendency to trigger coagulation, and the TAT levels remained low and comparable to initial samples and controls after incubation with whole blood. The generation of complement activation markers C3a and sC5b-9 was also reduced in the CNF_PEG_TiBALH samples compared to the control surfaces where the chamber model itself induces a weak complement activation, i.e., more or less identical to the initial samples. The anticoagulant effect is believed to, at least partly, be a result of residual lactic acid from the precursor, possibly causing lactic acidosis [42]. A recent publication has shown a very clear effect of pH on thrombin activity, where a decrease in one pH unit from 7.5 to 6.5 resulted in a reduced thrombin activity of about 65% [43]. Corresponding extrapolation down to pH 6, which was the actual pH of the CNF_PEG_TiBALDH surface, would result in a thrombin activity of less than 10%, which explains the inhibited thrombin generation as well as the subsequent TAT and clot formation. Similarly, it has been reported that one of the major complement inhibitors Factor I, together with its cofactor Factor H, has its optimal proteolytic activity at pH 6 [44]. Factor I cleaves C3b to iC3b, thereby preventing new C3 convertases to be formed, which results in discontinued complement activation. This is probably the reason why there is no complement activation at all in the TiBALDH samples, even though the blood model itself induces a low degree of activation. The various properties of the nanotitania-nanocellulose hybrids show potential for the design of specific surfaces for different applications. The coagulative properties of CNF_PEG_TiO_2_ and CNF_PEG_Captigel materials makes them suitable candidates for applications such as wound healing patches, possibly with the additional advantage of promoting tissue generation. CNF_PEG_TiBALDH, on the other hand, seems to prevent activation on the surface and has a wide range of uses where that type of reaction is not desirable, for example for drug delivery systems, catheters, and extracellular circuits.

Apart from being a temporary protection of the open wound, the clot also represents a scaffold for migration of immune cells, i.e., neutrophils and monocytes, during the healing process [45]. Activated platelets contribute both by being one of the main actors in the clot formation and by releasing chemokines and growth factors, which accelerates the recruitment and subsequent activation of immune cells to the damage area. It is therefore likely that platelet and coagulation activation are of utmost importance in the wound healing process, and materials that accelerate the coagulation will be very beneficial for efficient healing.

## 5. Conclusions

The effect of nanotitania-nanocellulose hybrid materials on human whole blood was investigated through an in vitro study. Two of the hybrids, CNF_PEG_TiO_2_ and CNF_PEG_Captigel, induced strong coagulation and platelet reduction but did not appear to cause any substantial complement activation. The strong blood clotting capacity of these two materials makes them promising candidates for wound dressings with the capacity to promote healing. The third hybrid material, CNF_PEG_TiBALDH, caused only minimal coagulation activation. In addition, the complement markers C3a and sC5b-9 remained at the same level as in the initial samples. The inhibitory effect on clot formation and complement activation of the CNF_PEG_TiBALDH material could make it suitable for applications where coagulation is undesired, such as in drug delivery systems and extracorporal circuits. This also shows the possibility of designing surfaces with specific functionalities by providing them with different types of nanotitania.

## Data Availability

Original data can be obtained from the corresponding author.
